# Supplementary material for: Expanding the Clinical Phenotype of 19q Interstitial Deletions: A New Case with 19q13.32-q13.33 Deletion and Short Review of the Literature
Source: Genes (Basel). 2022 Jan 24;13(2):212. doi: 10.3390/genes13020212 (PMC8872157; doi:10.3390/genes13020212)
Supplement: Supplementary file 1 [file genes-13-00212-s001.zip › genes-1459597-supplementary.pdf]

genes

MDPI

Supplementary Materials

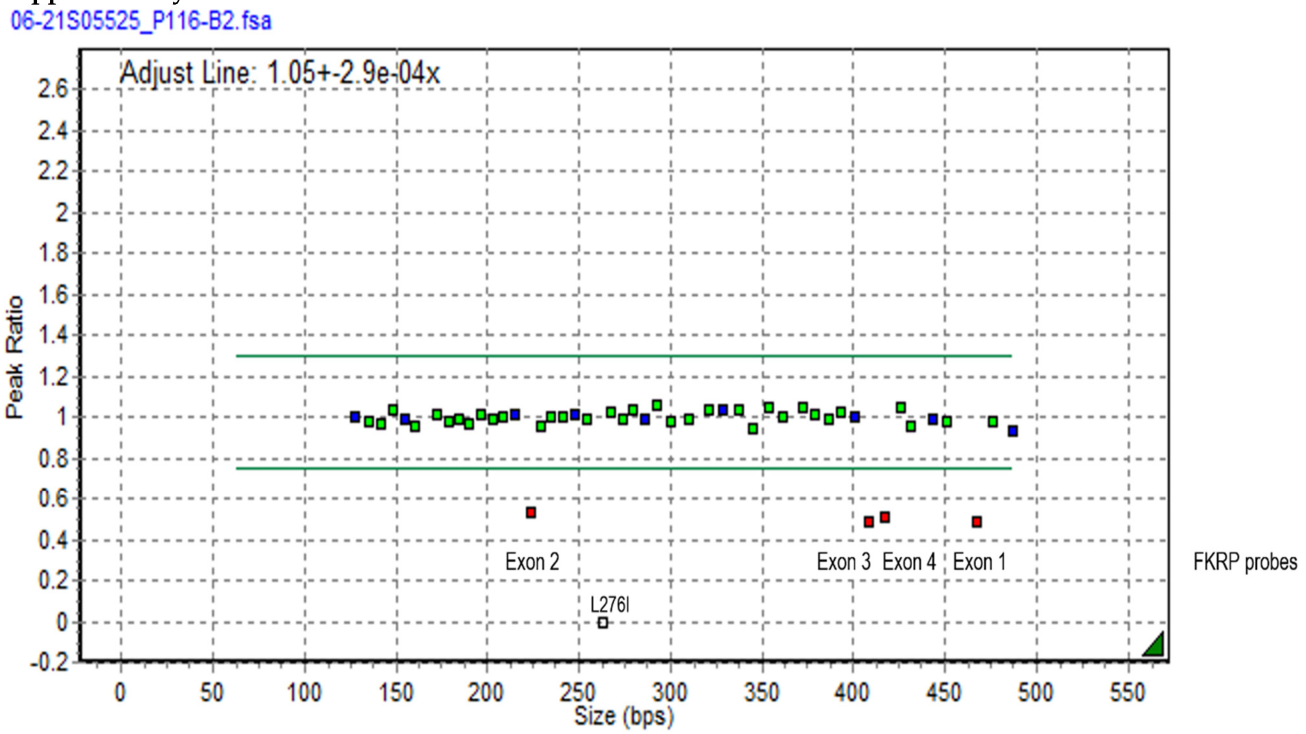

Figure S1. MLPA analysis of DNA from the patient. MLPA kit: MRC Holland, P116_B2, containing probes for each of the 4 exons of FKRP plus a specific probe for the recurrent L2761 mutation. Data analysis was made using Softgenetics Genemarker v 2.7.4. The red points indicate the heterozygous deleted exons; the recurrent L276I is absent (as expected) and so gives no signal.
